# Self-organization of chemoattractant waves in *Dictyostelium* depends on F-actin and cell–substrate adhesion

**DOI:** 10.1098/rsif.2016.0233

**Published:** 2016-06

**Authors:** Fumihito Fukujin, Akihiko Nakajima, Nao Shimada, Satoshi Sawai

**Affiliations:** 1 Department of Basic Science, Graduate School of Arts and Sciences, University of Tokyo, 3-8-1 Komaba, Meguro-ku, Tokyo 153-8902, Japan; 2 Research Center for Complex Systems Biology, Graduate School of Arts and Sciences, University of Tokyo, 3-8-1 Komaba, Meguro-ku, Tokyo 153-8902, Japan; 3 PRESTO, Japan Science and Technology Agency, Kawaguchi-shi, Saitama 332-0012, Japan

**Keywords:** chemotaxis, oscillations, mechanosensing, reaction–diffusion, *Dictyostelium*

## Abstract

In the social amoeba *Dictyostelium discoideum*, travelling waves of extracellular cyclic adenosine monophosphate (cAMP) self-organize in cell populations and direct aggregation of individual cells to form multicellular fruiting bodies. In contrast to the large body of studies that addressed how movement of cells is determined by spatial and temporal cues encoded in the dynamic cAMP gradients, how cell mechanics affect the formation of a self-generated chemoattractant field has received less attention. Here, we show, by live cell imaging analysis, that the periodicity of the synchronized cAMP waves increases in cells treated with the actin inhibitor latrunculin. Detail analysis of the extracellular cAMP-induced transients of cytosolic cAMP (cAMP relay response) in well-isolated cells demonstrated that their amplitude and duration were markedly reduced in latrunculin-treated cells. Similarly, in cells strongly adhered to a poly-l-lysine-coated surface, the response was suppressed, and the periodicity of the population-level oscillations was markedly lengthened. Our results suggest that cortical F-actin is dispensable for the basic low amplitude relay response but essential for its full amplification and that this enhanced response is necessary to establish high-frequency signalling centres. The observed F-actin dependence may prevent aggregation centres from establishing in microenvironments that are incompatible with cell migration.

## 1. Introduction

Cell movement and migration are often directed by self-generated diffusive extracellular signals; cells move according to the spatio-temporal profiles of molecules that are produced by themselves. In bacteria, chemotaxis towards excreted amino acids leads to the formation of complex colony patterns [1]. In mammary epithelium cells, self-generated subcellular gradient of autocrine EGF signals stimulate motility and polarization [2]. Similarly, a self-generated gradient of chemokine Sdf1 drives collective migration of lateral line primordium in zebrafish [3]. Directionality of cell movement also depends on mechanical parameters, such as surface rigidity and topography, confinement and adhesion [4]. F-actin formation in *Dictyostelium* is known to align along the nanoscale ridges on the surface substrate and induces biased directional movement [5]. Direction of fibroblast cell migration [6] as well as efficiency of neutrophil and macrophage migration depends on substrate stiffness [7,8]. Although it is becoming increasingly clear that physical and chemical cues play equally important roles in determining cell migration, how these two are mutually coordinated is less studied. In particular, how mechanical parameters influence synthesis and formation of a self-generated chemoattractant field has so far received little attention.

A classic example of self-generated signals in cell migration is found in the social amoeba *Dictyostelium*. Aggregation is mediated by chemotaxis towards travelling waves of extracellular cyclic adenosine monophosphate (cAMP) [9,10] that propagate across the cells at a periodicity of about 5–10 min [11,12]. The periodic signal is generated by the so-called cAMP relay response where an elevation of the extracellular cAMP level promotes further synthesis of cAMP [13] that is secreted to extracellular space [14] to excite neighbouring cells. Periodic changes in the cAMP level not only direct the chemotaxis of dispersed cells during the early stage of cell aggregation, but are also important in the later stage for cell migration [15,16] and gene expression [17]. By employing perfusion and live cell imaging of cytosolic cAMP, it has been demonstrated that the onset of collective pulses of cAMP is dictated by the stochastic cAMP pulses that are self-enhanced in the population [18–20] and that the oscillations require removal of extracellular cAMP by secreted phosphodiesterase [21]. These studies are largely compatible with a view that oscillations and waves of cAMP can be understood in the framework of reaction–diffusion systems [22] akin to the well-studied Belousov–Zhabotinsky reaction [23].

While the existing models that incorporated cell movement rules to the reaction–diffusion dynamics of cAMP have sufficiently explained the overall pattern formation of waves and cell streaming [24–27], the assumption that the cAMP relay response is more or less independent from cell movement may be an oversimplification in the light of heterogeneity and complexity of the cells and the surrounding environment. In this study, we address how the state of cytoskeleton influences emergence of the chemoattractant field by studying the population-level cAMP oscillations and the underlying single-cell level cAMP relay response under conditions that are inhibitory to F-actin. We demonstrate pharmacologically that amplification of cAMP necessary for the collective pulses is F-actin-dependent. Furthermore, we show that similar effect is observed in cells strongly attached to poly-l-lysine (PLL)-coated glass surface or cells treated with a PI3 kinase (PI3K) inhibitor. The present results suggest that a mutual regulation between chemoattractant field and F-actin may provide a means to prevent cells from migrating towards a deleterious environment.

## 2. Results

### 2.1. Collective cAMP oscillations are suppressed by latrunculin treatment

**Figure 1.**
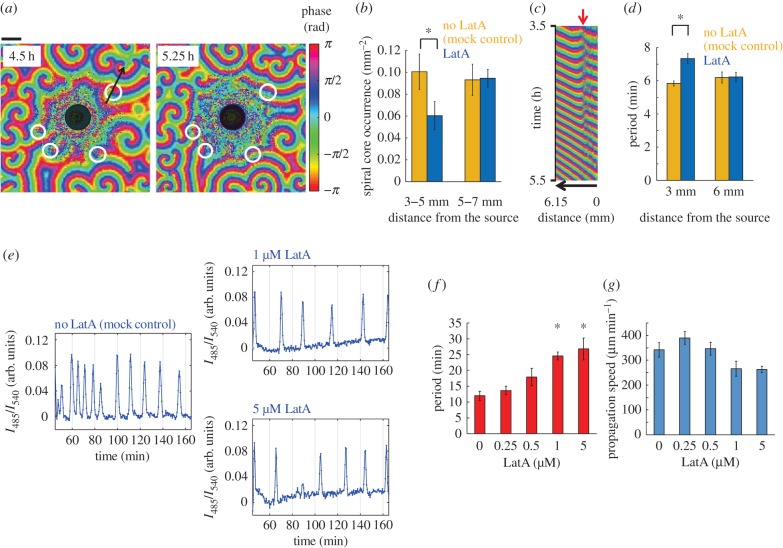
LatA treatment is inhibitory to population-level cAMP waves and oscillations. (*a*–*d*) Signalling centres near the LatA source (shaded centre circle) are extinguished as a result of wave competition. (*a*) Snapshots of darkfield wave oscillations observed in cells on agar at 4.5 h (left panel) and 5.25 h (right panel) after nutrient removal. The spiral wave centres (white rings) near the LatA source become entrained to waves from the periphery. Phase was extracted by wavelet transform and shown in colour. The scale bar is 3 mm. (*b*) The number of spiral cores decreases in regions closer to the LatA source. (*c*) A kymograph taken from a cross section (the black arrowed line in (*a*) left panel; 5 pixel wide average, 6.15 mm). The red arrow indicates a spatial phase singularity. (*d*) Mean period of the oscillations is lengthened near the LatA source. Error bars are standard error. Asterisks indicate statistical significance between control and LatA-applied populations (Welch *t*-test *p < 0.005; *n* = 6 independent samples). (*e*) Representative time series of the spatially averaged fluorescence intensity ratio *I*_485_/*I*_540_ of cells expressing the cAMP sensor Epac1camps treated with (right panels) or without (left panel) LatA (see Materials and methods). (*f*) Dose dependence of the interval between pulses indicates that occurrence of the population-level pulses is suppressed by LatA treatment. (*g*) Wave propagation speed is slowed down moderately. Error bars indicate standard error (from 0 to 5 µM, *n* = 3, 3, 5, 3, 4 independent samples). Asterisks indicate statistical significance between control and LatA-treated populations (Welch *t*-test *p < 0.005) (*f*,*g*).

We first examined the cAMP oscillations in *Dictyostelium* cell monolayer under the influence of latrunculin A (LatA)—an inhibitor of actin polymerization. Starved cells were plated on agar with a small round well (6.5 mm diameter) where 5 µM LatA was added at *t* = 2.5 h to form a concentration gradient (see Material and methods). Because fluorescence imaging becomes suboptimal in the presence of agar sheet, here we instead employed darkfield optics to capture spatio-temporal changes in the optical density that serve as a surrogate for the cAMP waves [11,28]. Waves of spiral form develop over the course of several hours after nutrient removal (figure 1*a*, left panel). The optical density waves were less visible near the LatA source as expected owing to cells becoming less motile. Because the optical density reflects a periodic shape change that occurs in sync with the cAMP waves, the present analysis cannot discriminate between decrease in the level of cAMP and that of a cell shape change. An estimate from the measurement of diffusion of a fluorescent probe indicates that, by *t* = 8 h, the entire field was saturated with LatA concentrations of approximately 0.25–1 µM (electronic supplementary material, figure S1) consistent with the timing by which all cells halted aggregation. Despite these limitations, there was still a notable feature during the first 7 h in regions where waves were visible. Close inspections revealed that the centres of spiral waves that initially appeared in regions close to the LatA reservoir eventually vanished (figure 1*a*, right panel). As a result, the final number of spiral centres per area were fewer in regions closer to the latrunculin pool compared with that in the outer region (figure 1*b*). A kymograph (figure 1*c*) shows a clear invasion of wave territory—after rounds of wave collision, waves propagating from the outer region eventually took over the waves from the regions closer to the centre. Such entrainment of oscillations in excitable and oscillatory media often occurs by difference in the wave periodicity. The point of wave collision gradually moves towards the signalling centres with low frequency, because after wavefronts collide and annihilate, the next wavefront from the faster oscillating region will advance more than that from the slower-oscillating region before colliding again. Indeed, while the mean periodicity of the waves in the outer region was about 6 min thus relatively unaffected (figure 1*d*), the periodicity in the intermediate areas increased to about 7.4 min (figure 1*d*; 3–5 mm). Cells under a long-time exposure to LatA were still able to recover a motile cell shape after wash out (electronic supplementary material, figure S2). These observations suggest that inhibition of actin polymerization increased the periodicity of cAMP waves and influenced the competition between signalling centres [29].

To elucidate the effect of LatA more quantitatively, we measured the oscillations of cAMP in cells expressing the cAMP sensor Epac1camps [30]. Here, 4.5–5 h starved cell suspension was plated on a coverslip and observed without perfusion under high-magnification lens. Under this condition, oscillations of cAMP occur (figure 1*e*, left panel) owing to the accumulation of extracellular phosphodiesterase that resets the extracellular cAMP level after each pulse [21,31]. Figure 1*e* (right panels) shows a representative time series of cells treated with 1 or 5 µM LatA. Here, the mean fluorescence ratio was plotted, because the phase difference across the field of view was small. The peak-to-peak intervals were markedly elongated in LatA-treated cells compared with non-treated cells (figure 1*e*). On the other hand, the amplitude and the form of each pulse were only moderately perturbed by LatA treatment. We have repeated these experiments for several LatA concentrations and found that the period of synchronized cAMP pulses increased in a dose-dependent manner (figure 1*f*). The wave propagation speed was also somewhat lowered (figure 1*g*). These results are consistent with the increase in the period of the optical density waves on agar.

### 2.2. The amplitude and the duration of cAMP-induced increase in cytosolic cAMP are F-actin-dependent

**Figure 2.**
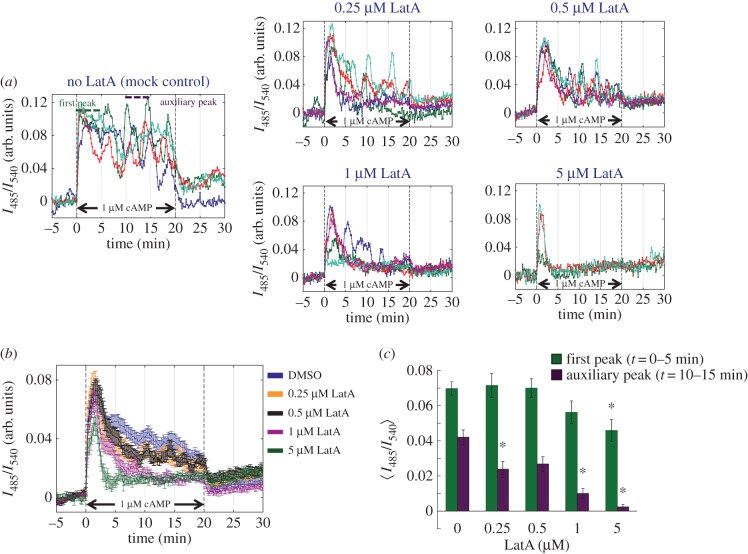
Persistence of the relay response to micromolar cAMP at the single-cell level is markedly reduced in LatA-treated cells. (*a*) Representative time series of the response to 1 µM cAMP stimulation in well-isolated Epac1camps expressing cells. No LatA control (left panel) and LatA (middle and right panels; 0.25, 0.5, 1 and 5 µM LatA). Colours indicate different cells in the field of view. (*b,c*) Average of the relay response (colours indicate LatA dose) (*b*) and dose–response relationship of the response maxima sampled at *t* = 0–5 min (first peak; green) and *t* = 10–15 min (auxiliary peak; purple) (*c*). Error bars are standard error. Asterisks indicate statistical significance between control and LatA-treated cells (Welch *t*-test *p < 0.005; *n* = 42, 22, 15, 19, 17).

There are two main properties that determine the peak-to-peak interval of synchronized cAMP pulses. One is the rate of the cAMP-induced transient of cAMP synthesis and secretion which depends strictly on the accumulation of extracellular cAMP [18,20]. The other is the time it takes for the cells to recover responsiveness after removal of the stimulation; i.e. time to ‘de-adapt’ [32]. To clarify these aspects, we measured the cAMP-induced transient in cytosolic cAMP (hereafter refer to as cAMP relay response) in well-isolated single cells. To generate a step increase in the extracellular cAMP concentration, a microfluidics chamber (electronic supplementary material, figure S3*a*) was used to facilitate perfusion and rapid exchange of buffer within 2 s (electronic supplementary material, figure S3*b*). In the absence of LatA, the sustained oscillatory response was observed (figure 2*a*, left panel) for 1 µM cAMP stimulation consistent with an earlier observation in a large open top chamber [18], indicating that cell confinement in the present chamber did not affect the overall cAMP response property (see also [20]). In cells treated with LatA, the response to 1 µM cAMP stimulation attenuated faster, and there was an overall reduction in the amplitude. At 0.25 and 0.5 µM LatA (figure 2*a*, middle and right top panels), the effect on the initial response (*t* = 0–5 min) was marginal, whereas the magnitude of the prolonged response (*t* = 10–15 min) was reduced. At higher concentrations of LatA (greater than or equal to 1 µM), the prolonged response was severely hindered (figure 2*a*, middle and right bottom panels). We did not observe a notable change in the intervals of auxiliary peaks during the prolonged response, if they appeared. Although these features were somewhat cryptic at the single-cell level owing to cell–cell variability of the response, the overall trend was evident when averaged (figure 2*b*,*c*). The magnitude of the major first transient was slightly reduced compared with the untreated cells (figure 2*c*; green), whereas the persistent response was reduced by more than 50% (figure 2*b*,*c*; greater than 0.25 µM; purple). The dose–response (figure 2*c*) was close to the concentrations of LatA required to inhibit actin polymerization [33]. The observed cells were not irreversibly damaged as when washed free of the drug for about 10 min the response was fully recovered (electronic supplementary material, figure S4).

**Figure 3.**
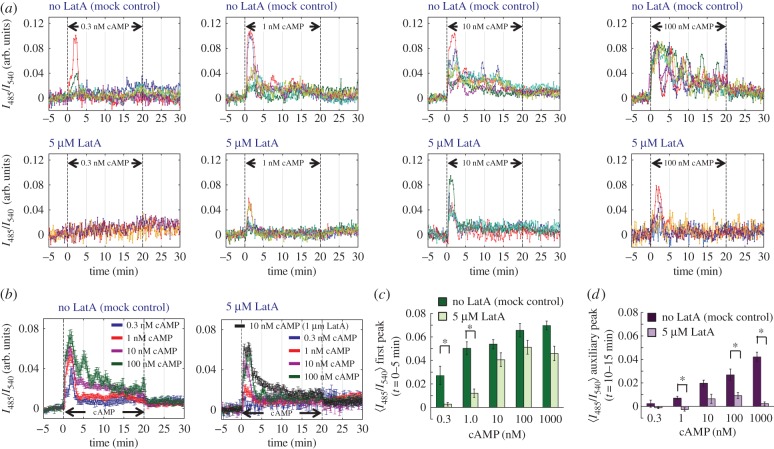
The initial peak of the relay response to nanomolar cAMP stimulus was severely suppressed in LatA-treated cells. (*a*) cAMP relay response in well-isolated single cells (controls and LatA-treated cells; top and bottom panels). Extracellular cAMP was applied from *t* = 0–20 min (dashed lines) at the concentrations of 0.3, 1, 10 and 100 nM (from left to right). (*b*–*d*) The average time series (*b*) and dose–response relationship of the response maxima sampled at *t* = 0–5 min (first peak; green) (*c*) and *t* = 10–15 min (auxiliary peak; purple) (*d*). Error bars are standard error (controls *n* = 18, 32, 51, 15, 42 and LatA-treated cells *n* = 4, 15, 13, 19, 17). Asterisks indicate statistical significance between control and LatA-treated cells (Welch *t*-test *p < 0.005). The 1 µM cAMP data from 5 µM LatA-treated cells in figure 2 are shown here for comparison.

We next tested the cAMP relay response of cells under 5 µM LatA treatment to various concentrations of extracellular cAMP (figure 3*a*). In contrast to 1 µM stimulation, the response to less than 10 nM cAMP was short-lived and adapted perfectly as shown earlier [18,34] (figure 3*a* top panels). In LatA-treated cells, the peak magnitude of the response was diminished markedly to about 10–20% of the untreated cells (figure 3*a* bottom panels; figure 3*b,c,* 0.3 and 1 nM). For 10 nM and higher concentrations of cAMP, the initial peak of the response was less marked, reaching approximately 80% of the untreated cells (figure 3*b*,*c*, 10 and 100 nM). On the other hand, the amplitude of the auxiliary response was reduced significantly to about 5–20% of the full response (figure 3*d*, 10 and 100 nM). Figure 3*c*,*d* summarizes these features by plotting the average maxima of the response at *t* = 0–5 min (figure 3*c*) and *t* = 10–15 min (figure 3*d*). The results suggest that F-actin acts positively to elevate the level of cytosolic cAMP and that this is crucial for amplification of the single-peaked response to nanomolar extracellular cAMP stimulus as well as for the persistent response to micromolar extracellular cAMP.

**Figure 4.**
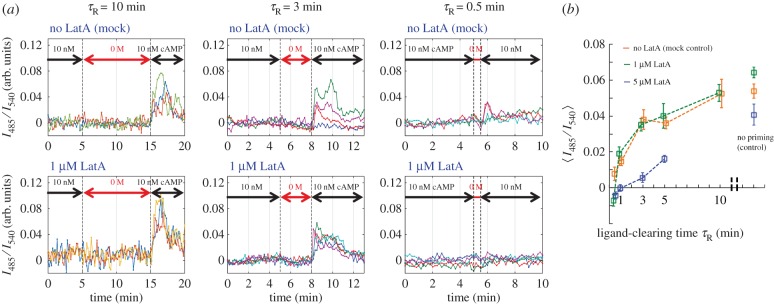
The kinetics of deadaptation was not affected by LatA treatment. Cells exposed to persistent stimulations for more than 20 min were washed with buffer for indicated time *τ*_R_ then stimulated again with the same concentration of cAMP. (*a*) Representative time course of the response after washing in mock-treated (top panels) and 1 µM LatA-treated cells (bottom panels). (*b*) The recovery of the average response as a function of washing time *τ*_R_. The magnitude and the rate of recovery were not perturbed by 1 µM LatA treatment. Error bars are standard error (controls *n* = 8, 20, 22, 45, 9, 51 and 1 µM LatA *n* = 5, 10, 20, 15, 15, 34 for *τ*_R_ = 0.5, 1, 3, 5, 10 min and no priming, respectively; for 5 µM LatA *n* = 5, 6, 7, 5, 13 for *τ*_R_ = 0.5, 1, 3, 5 min and no priming, respectively).

The length of the refractory period is the other important determinant of the periodicity. Cells responded to repetitive stimuli at 5 min intervals; however, the response attenuated when the stimulus intervals were less than 3 min [18,20]. Earlier biochemical studies [32] have shown that the duration of the refractory period in the cAMP relay response was about 5–10 min long. Following the same stimulus scheme [32], we characterized the time required for pre-stimulated cells to recover responsiveness (i.e. to ‘de-adapt’) at the single-cell level. Cells were first primed by exposure to 10 nM cAMP, then cleared of extracellular cAMP with buffer flow for time *τ*_R_ before switching back to the flow containing 10 nM cAMP (figure 4*a*). Note that, for the analysis, an intermediate dose of LatA (1 µM) was employed where the periodicity of the population-level oscillations was increased markedly, whereas the first peak of the cAMP relay response to nanomolar stimulus at the single-cell level was comparable to the mock-treated cells (figure 3*b*, right panel). Figure 4*b* shows a representative time series of such experiments. We found that the magnitude of the response became almost undetectable when the wash time was 1 min or less. The response gradually recovered as the wash time was increased and reached approximately 80% of the full response after 5 min of washing (figure 4*b*). The slope or the *x*-axis crossing of the curve (figure 4*b*) showed no significant difference between treated and non-treated cells, and agreed well with the earlier population-level biochemical assays (fig. 4 in [32]). While 1 µM LatA treatment almost doubled the period of the population-level oscillations (figure 1*f*), the same dosage of LatA did not perturb duration of refractoriness. For 5 µM LatA-treated cells, the maximal response was significantly reduced making it difficult to compare the recovery kinetics with the control (figure 4*b*).

### 2.3. The amplitude and the duration of cAMP-induced increase in cytosolic cAMP are suppressed by strong cell–substrate adhesion or PI3K inhibition

**Figure 5.**
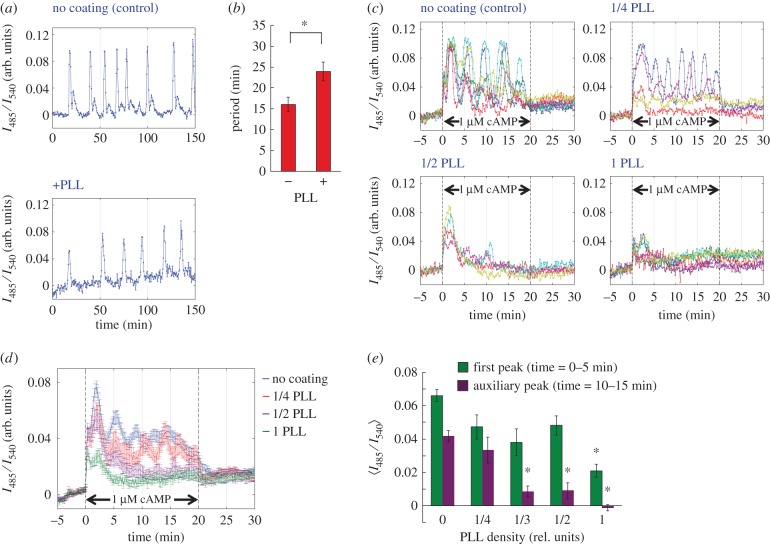
The collective oscillations and the cAMP relay response were suppressed in cells attached to PLL-coated coverslips. (*a*) The cAMP oscillations in cell populations on a non-coated (top panel) or a PLL-coated coverslip (bottom panel). 1 PLL unit refers to a chamber coated with 0.5 mg ml^−1^ PLL solution. (*b*) The periodicity of the population-level cAMP oscillations. Error bars are standard error (*n* = 6 for both non-coated and PLL-coated). The asterisk indicates statistical significance between non-coated and PLL-coated surface (Welch *t*-test *p < 0.005). (*c–e*) The relay response of single cells on coverslip surfaces with varying degree of PLL coating. Time series of individual cells (colour indicates different cells in the field of view) (*c*), average time series (*d*) and response maxima sampled at *t* = 0–5 min (first peak; green) and *t* = 10–15 min (auxiliary peak; purple) (*e*). Error bars are standard error (*n* = 31, 15, 6, 17, 12; in the order of no PLL to 1 PLL density). Asterisks indicate statistical significance between non-coated and PLL-coated surfaces (Welch *t*-test *p < 0.005).

Results from the LatA-treated cells suggest that the cAMP relay response may be sensitive to mechanical perturbations. To explore this aspect, we studied the effect of coating the glass substrate with PLL. Owing to the positive charge, PLL promotes cell–substrate adhesion strong enough to hinder random cell migration (electronic supplementary material, figure S5). An earlier work has shown that cells on a PLL-coated surface rapidly clumped together to form tiny clusters instead of streaming into a large mound [35]. Moreover, for reasons not understood, the overall level of F-actin is known to decrease in cells adhered to PLL-coated glass [35]. Figure 5*a* shows a representative time course of the population-level cAMP oscillations in cells attached to a PLL-coated glass coverslip. The effects on the amplitude and the period of the oscillations were similar to those observed in LatA-treated cells. The amplitude was slightly reduced (figure 5*a*), and the period was lengthened by about 30% (figure 5*a*,*b*). As expected, in well-isolated single cells attached to a PLL-coated glass surface, the peak magnitude as well as persistence of the relay response was reduced in a dose-dependent manner (figure 5*c–e*).

**Figure 6.**
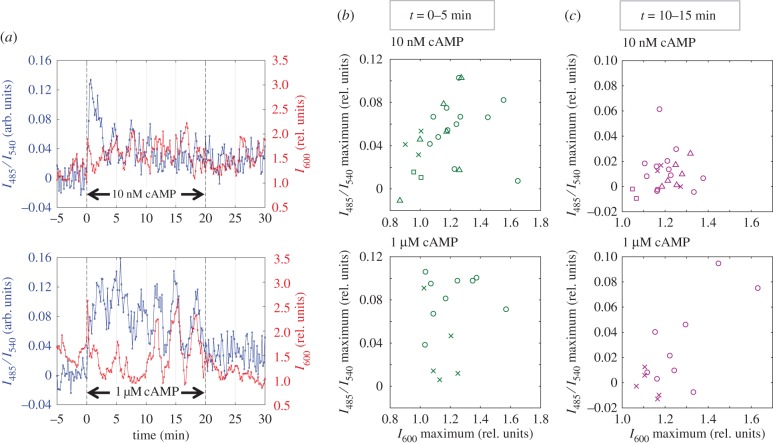
The cAMP relay and the F-actin response are correlated at the single-cell level. Data were acquired by confocal microscopy from cells co-expressing Epac1camps and LifeactRFP. (*a*) Representative time series of cells exposed to 10 nM cAMP (top panel) and 1 µM cAMP (bottom panel). *I*_600_ is the average LifeactRFP fluorescence intensity of a membrane region relative to that of the intracellular region. Peaks of LifeactRFP occasionally coincided with those of cytosolic cAMP. (*b*,*c*) Scatter plots of response maxima at *t* = 0–5 min (*b*) and *t* = 10–15 min (*c*) for stimulus concentration of 10 nM (top panels) and 1 µM cAMP (bottom panels). Plots are compilation of data from controls (circle), 1 µM LatA-treated cells (triangle), 5 µM LatA-treated cells (square) and cells on 1PLL-coated surface (cross).

To further elucidate the quantitative relationship between the levels of cytosolic cAMP and actin polymerization, we measured cells expressing both Epac1camps and LifeactRFP by confocal microscopy. Upon exposure to 10 nM or 1 µM cAMP, the overall change in the cytosolic cAMP level appeared similar to the aforementioned measurements based on epifluorescence [18]. There was no discernible spatial heterogeneity (electronic supplementary material, figure S6) indicating that, at the time resolution of the present imaging, cAMP rapidly equilibrates within the cytosol. LifeactRFP fluorescence, on the other hand, appears as bright patches on the plasma membrane that appear sporadically even before stimulation [33]. Upon cAMP exposure, cortical fluorescence of LifeactRFP (*I*_600_) increased and peaked within 10 s (figure 6*a*; *t* ∼ 0–1 min). After about 2–4 min, auxiliary peaks of LifeactRFP fluorescence appeared. These spikes in LifeactRFP were not markedly in phase with the peaks of the cytosolic cAMP level (figure 6*a*, top panel); however, there were some instances where they coincided (figure 6*a*, bottom panel). Figure 6*b* is a scatter plot that compares the magnitude of the first peaks of LifeactRFP fluorescence and that of the cytosolic cAMP level. Whereas the two show a strong correlation for 10 nM cAMP stimulation (figure 6*b*, top panel), the correlation was weak for 1 µM cAMP stimulation (figure 6*b*, bottom panel). By *t* ∼ 10–15 min, the cAMP relay response to 10 nM cAMP stimulus more or less adapted, and the correlation between cAMP relay and F-actin was weak (figure 6*c*, top panel). At 1 µM cAMP stimulation, the response in cytosolic cAMP was more persistent, and there was a higher correlation (figure 6*c*, bottom panel). These results agree well with the property of LatA-treated cells. For nanomolar cAMP stimulus, the magnitude of the cAMP relay response (*t* = 0–5 min) depended strongly on F-actin, whereas, for micromolar cAMP stimulus, F-actin dependency was more notable for the persistent response (*t* = 10–15 min; figure 3*b–d*).

**Figure 7.**
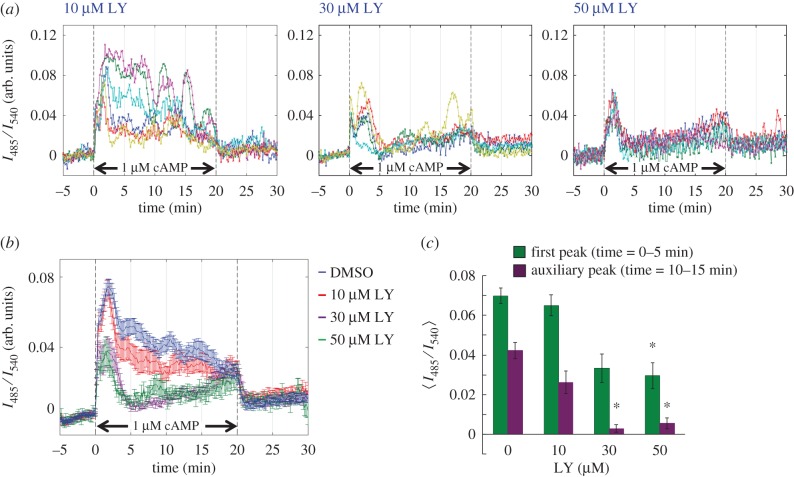
The cAMP relay response is attenuated in LY-treated cells. (*a*) Representative time series of the relay response to 1 µM cAMP in cells exposed to 10, 30 and 50 µM LY (panels from left to right, top to bottom). (*b*,*c*) Average time series (*b*) and response maxima sampled at *t* = 0–5 min (first peak; green) and *t* = 10–15 min (auxiliary peak; purple) (*c*). The mock-treated data from figure 2*b* are shown in (*b*) for comparison. Error bars are standard error (*n* = 31, 15, 6, 17, 12; in the order of no PLL to 1PLL density). Asterisks indicate statistical significance between non-coated and PLL-coated surfaces (Welch *t*-test *p < 0.005).

The signal transduction cascade that leads to the cAMP relay response has a large overlap to that for the chemotactic response [36]. This includes receptor-mediated activation of PI3K which has been shown to be essential not only for actin polymerization but also for activation of the adenylyl cyclase ACA [37]. It has been suggested that a positive feedback loop mediated by Ras–PI3K–F-actin amplifies the leading edge forming signals during both spontaneous cell shape change as well as chemotactic migration [38]. An earlier study has shown that latrunculin-treated cells exhibit much reduced translocation of PI3K and Akt/PH to the plasma membrane upon cAMP stimulation [39,40]. In assays for directional sensing towards artificial cAMP waves, the magnitude of Ras activation at the leading edge has also been shown to decrease by latrunculin treatment [9]. Consistent with these observations, in cells treated with LY294002 (LY), an inhibitor of PI3K, we observed a marked reduction in the first peak as well as the auxiliary response peaks. At 10–30 µM LY treatment, the initial response was almost unaffected, whereas the prolonged response observed for *t* = 10–15 min was markedly reduced (figure 7*a*,*b*). At 50 µM LY, the initial response peak was reduced by about 50% (figure 7*c*). The effective dosage of LY agrees well with the concentration necessary to inhibit PI3K and other downstream components. Although the overall effect of the LY on the cAMP relay response is similar to latrunculin, there was one dissimilarity. At 30 and 50 µM LY, there was a tendency for the cytosolic cAMP to gradually elevate (figure 7*b; t* = 15–20 min) which was never observed in latrunculin-treated cells. A similar but even more exaggerated response has been reported in earlier biochemical assays of PI3K1/2 knockout cells, thus may indicate that PI3K plays a role also in adaptation of adenylyl cyclase [37]. Because pharmacological inactivation of F-actin or PI3K did not completely extinguish the cAMP relay response, taken together with the fact that membrane translocation of PH domain of CRAC necessary for ACA activation still occurs in LatA-treated cells [41], the present data suggest that PI3K and F-actin are dispensable for the basic response but essential for the amplification of cAMP.

## 3. Discussion

The present results suggest that F-actin plays a critical role in the amplification of extracellular cAMP and to initiate the population-level oscillations. At the onset, the occurrence of the collective bursts of cAMP synthesis/secretion is determined by the accumulation of (sub)nanomolar extracellular cAMP [18]. Conditions that decrease extracellular cAMP accumulation such as mild inhibition of cAMP synthesis by caffeine or dilution of secreted cAMP in a perfusion chamber are known to increase the oscillation period [18,42]. This is consistent with our observations that the period of collective oscillations was markedly lengthened by 5 µM LatA treatment (figure 1*e*)—the same dosage that impaired the cAMP relay response to a nanomolar cAMP stimulus (figure 3*a*). Note however that the periodicity was also lengthened at 1 µM LatA (figure 1*e*) which abolished the persistent component only (figures 2*a* and 3*b*). Because the cells transiently experience about 1 µM cAMP at the peak [43], the non-adaptive component of the response to several 10–100 nM cAMP may help cAMP to be fully amplified so as to render the oscillations self-sustainable. It is known that the cAMP relay and other chemotactic responses are mediated by two receptors: CAR1 and CAR3 [34]. The binding affinities to cAMP are about 20 and 200 nM for CAR1 and 30 and 500 nM for CAR3, respectively. The majority of CAR3 is in the low affinity state, whereas a large percentage of CAR1 is in the high affinity state [44]. Cells expressing CAR1 but not CAR3 showed a more persistent cAMP relay response than wild-type cells, irrespective of the dose of cAMP [45]. In cells expressing CAR3 but not CAR1, the cAMP relay was reduced in magnitude with no auxiliary peaks [34], and the waves had a long periodicity [46]. These phenotypes of the CAR1-null cells are remarkably similar to those of the LatA-treated cells. Taken together with the cAMP dose-dependence of the present data, we speculate that the relay response during the collective oscillations is a superposition of outputs from two pathways; a CAR1-mediated persistent response that is F-actin-dependent combined with a CAR3-mediated response that is F-actin independent and more perfectly adapting. Similarly, strong cell–substrate attachment had a deleterious effect on the cAMP oscillations and relay. A previous study [35] has shown that cells on a PLL-coated substrate have decreased amount of polymerized actin and can only form small aggregates. The present results suggest that cells under such conditions may have formed aggregates simply by accretion without cAMP waves and chemotaxis similar to *acaA* knockout cells that are forced to differentiate by overexpressing protein kinase A. Similarly, LY is known to decrease the level of F-actin [47]; however, the relay response in LY-treated cells was distinct from that observed under LatA treatment. It may be that cells were exhibiting an additive effect of lowering F-actin and inhibiting PI3K and potentially other enzymes.

Our results suggest that F-actin either enhances the synthesis of cAMP or suppresses its degradation or secretion. Inactivation of ACA is immediate after clearing of extracellular cAMP, thus the kinetics of decrease in the cytosolic cAMP level after release from the stimulus is expected to be largely dictated by the rate of degradation and secretion. Because the rate of decrease in the cytosolic cAMP level after stimulus removal appears almost unaltered in LatA-treated cells (figure 2*b*; *t* = 20–22 min), it is likely that production of cytosolic cAMP was perturbed not degradation/secretion. This view is also consistent with the fact that the measured maximal peak intensity of the relay response was strongly correlated with the time derivative, i.e. the rate of increase in the level of cytosolic cAMP (electronic supplementary material, figure S7). cAMP in the aggregation stage *Dictyostelium* cells is synthesized by the plasma membrane-bound adenylyl cyclase ACA [48,49]. The cytosolic cAMP transients measured by the fluorescence resonance energy transfer (FRET) probe were completely abolished by application of a specific inhibitor of the adenylyl cyclase ACA [18]. The estimated amount of cAMP secreted from the cytosol can fully account for the amount of cAMP in the extracellular space [18]. These lines of evidence point to a possibility that ACA remains activated when coupled to actin cytoskeleton and that this forms a part of the positive feedback loop required for the excitability and the population-level organization of the cAMP signalling dynamics.

The present results are in agreement with earlier biochemical assays that have shown a decrease in total cAMP synthesis in LatA-treated cell populations [50]. Expression of a mutant form of actin Y53A that disrupts actin cytoskeleton inhibited ACA activation [51]. These assays were performed on total cAMP and did not discriminate between the cytosolic and other pools of cAMP. In fact, the decrease in cAMP has been attributed to cAMP in exocytic vesicles based on the evidence that vesicular accumulation of ACA was hampered in cells treated with latrunculin and cells expressing constitutively active ACA [50] or Y53A-actin [51]. The present results, however, demonstrated a reduction in the cytosolic cAMP pool. Because this study focused on the onset of cAMP signalling, we employed cells that are relatively early into starvation when ACA is still uniformly distributed in the plasma membrane. One should note that secretion of cAMP is immediate and constitutive [14] and does not require an exocytic pathway [52]. Adenosine triphosphate-binding cassette (ABC) transporters that carry cAMP across the plasma membrane are well known in mammalian cells, and a recent study has identified an ABC transporter AbcB3 [53] as a potential cAMP efflux pump in *Dictyostelium*. Apart from the acute effect of F-actin inhibition, coronin A which is believed to be associated with cortical F-actin is necessary to initiate the expression of ACA [54] implicating an additional layer of complexity in the developmental context. Future studies should address how the coupling between F-actin, ACA localization and cAMP dynamics depends on the developmental stage.

What is the biological role of the F-actin dependency? The low F-actin level that reduced the relay response is normally not attained in a standard aggregative laboratory condition. Therefore, we propose that the F-actin dependence of the relay response may provide a means to remotely sense and avoid adverse conditions for aggregation. A natural habitat of *Dictyostelium* such as soil is likely abundant in actin inhibitory molecules such as phalloidin and cytocharasins secreted by fungi. When under the influence of such toxins or adherent mucus surfaces that obtrudes cell movement, our results predict that cells that are less disturbed should in principle be able to self-generate cAMP more efficiently. According to the general property of excitable systems, wave territories become entrained to signalling centres, i.e. centres of target or spiral waves that emit cAMP pulses at the highest frequency. The pacemaker regions that survive this competition are expected to be farther away from the toxins and other physical conditions non-permissive to actin polymerization. Such a mechanism should facilitate cells from executing futile migration towards a microenvironment undesirable for aggregation and later morphogenetic movements.

From this work and others, it is becoming increasingly clear that many of the self-organized waves in living cells are intricately linked to the cytoskeletal machineries [55]. The spatio-temporal wave patterns of *Escherichia coli* Min protein depend on the surface [56] and compartment geometry [57] and involve polymerization of Min protein that are coupled to actin-like FtsZ and other cell division machineries. The phosphatidylinositol waves in the ventral side of the plasma membrane in *Dictysotelium* and waves of Rac activation in neutrophils are also strongly dependent on F-actin [58,59]. Propagation of curvature waves along the edge of *Dictyostelium* cells are known to stop when attaching to the surface [60]. These examples point to universality of mutual coupling between reaction–diffusion type self-organization and the mechanics of active matters [61,62] as the physical basis of cellular organization. As for the multicellular context, it would be interesting to see whether such mechanical coupling is in operation in self-organizing waves in embryonic development [63].

## 4. Material and methods

### 4.1. Cells and sample preparation

*Dictyostelium discoideum* AX4 cells constitutively expressing the cAMP sensor Epac1camps [18,30] alone or together with Lifeact [64,65] fused to mRFPmars (LifeactRFP) were employed. All cells for live cell imaging were grown as previously described [31,66]. In addition, 10 µg ml^−1^ G418 and 60 µg ml^−1^ hygromycin B were added appropriately for selection. Exponentially growing cells were washed twice and suspended in 1 ml developmental buffer (DB; 6 mM KH_2_PO_4_, 4 mM Na_2_HPO_4_, 2 mM MgSO_4_, 0.2 mM CaCl_2_, pH 6.5) at a cell density of 2.0 × 10^7^ cells ml^−1^ and shaken for 4.5–5.5 h at 22°C except for darkfield imaging where cells were plated immediately after nutrient removal. For darkfield observation, cells were plated on agar plates with a centre hole for LatA reservoir. For FRET observation of cell populations, either a glass-bottom dish (LatA) or a coverslip with a frame seal (PLL) was used. To measure the cAMP relay response at the single cell level, Y-shaped channel made of polydimethylsiloxane was used together with a pair of syringe pumps for perfusion. See the electronic supplementary material methods for details.

### 4.2. Image acquisition and analysis

For darkfield observations, at 2.5 h from the beginning of timelapse recording, 75 µl DB containing 5 µM LatA was applied to the hole. For mock control, DB containing 1% DMSO (the equivalent in the LatA solution) was used. To obtain phase information, wavelet transform was applied to averaged time series of 4 × 4 pixel regions in the raw data. Morlet function 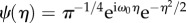 with *ω*_0_ = 6 [31] was used. The angular variable of the dominant frequency was extracted as described earlier [58]. For time series of FRET data, the average fluorescent intensities from the cell masks at 485 nm (*I*_485_) were divided by those at 540 nm (*I*_540_). For FRET data, the mean values between *t* = −5 to 0 min were subtracted in the data plots. For cAMP relay data on PLL-coated coverslip, cells that appear compromised in membrane integrity (17% for 0.5 PLL and 33% for 1 PLL) were excluded from analysis. Image analysis was performed using Matlab and ImageJ. To obtain LifeactRFP time series, the cell edge (1 µm width) was masked as a region of interest, and the ratio of its mean intensity and the intensity of the inner cytosolic region was calculated.

## Supplementary Material

Supplementary Materials

## Data accessibility

Dataset for figures are available on Figshare: https://dx.doi.org/10.6084/m9.figshare.3362716.
