# Supplementary material for: Self-organization of chemoattractant waves in Dictyostelium depends on F-actin and cell–substrate adhesion
Source: J R Soc Interface. 2016 Jun;13(119):20160233. doi: 10.1098/rsif.2016.0233 (PMC4938087; doi:10.1098/rsif.2016.0233)
Supplement: Supplementary Materials [file rsif20160233supp1.pdf]

Supplementary materials for

Self-organization of chemoattractant waves in *Dictyostelium* depends on F-actin and cell-substrate adhesion.

Fumihito Fukujin[1], Akihiko Nakajima[2], Nao Shimada[1] and Satoshi Sawai[1,2,3]

[1]Department of Basic Science, [2]Research Center for Complex Systems Biology, Graduate School of Arts and Sciences, University of Tokyo, 3-8-1 Komaba, Meguro-ku, Tokyo 153-8902, Japan. [3] PRESTO, Japan Science and Technology Agency, Kawaguchi-shi, Saitama 332-0012, Japan.

Author for correspondence:
Satoshi Sawai
e-mail: cssawai@mail.ecc.u-tokyo.ac.jp

This file contains:

Supplementary Materials and Methods,
Supplementary Figure S1,
Supplementary Figure S2,
Supplementary Figure S3,
Supplementary Figure S4,
Supplementary Figure S5,
Supplementary Figure S6,
Supplementary Figure S7,

Supplementary References.

Supplementary Materials and Methods

Reagents

cAMP-Na (A6885, Sigma) was dissolved in water at 100 mM, aliquoted and stored at -30 °C as a master stock. The working solution of cAMP was obtained by diluting the master stock solution with DB to final concentration of 10 μM or 0.1 μM. LatA (125-04363, Wako) was dissolved in dimethyl sulfoxide (DMSO, 047-29353 Sigma) at 1 mM and stored at -30 °C. Poly-L-lysine (PLL) was obtained as a solution (P4832, Sigma) and stored at 4 °C.

Plasmid construction

For construction of the LifeactRFP expression plasmid, an RFP expression vector for hygromycin selection pHygEX-mRFPmars was first constructed from the PHcracRFP expression plasmid (a kind gift of Dr. Richard Firtel) [1] by introducing a new flanking *Bgl*II site to remove PHcracRFP and introduce a new *Cla*I/*Xho*I cloning site. Synthetic oligonucleotides for sense- and antisense strands of codon-optimized Lifeact [2] with flanking *Cla*I sites were annealed and ligated to *Cla*I linearized pHygEX-mRFPmars vector. The obtained LifeactRFP expression plasmid was introduced to Epac1camps/AX4 [3] by electroporation according to a standard procedure [4] and clones were selected under 60 μg/mL Hygromycin B.

Sample preparation and stimulus delivery

For darkfield observation of population-level oscillations, growing cells were washed and plated immediately at approximately 1/4 monolayer cell density (1.67×10^5 cells/cm^2) on a 1% DB–agar plate (3 mm thick, 60 mm diameter) with a 6.5 mm diameter hole in the center. The hole was made by pulling out an agar disc using a Pasteur pipette. The cells were allowed to settle for 25 min before the supernatant was removed. The plates were then allowed to dry for 15 min in a sterile hood before starting timelapse observation.

For high magnification observation of population-level oscillations in LatA-treated cells, starved cell suspension was 40x diluted with DB to final volume of 250 μL and plated on a glass bottom dish (14 mm diameter; P35G-0-14-C, MatTek) so that the cell density is approximately 1/4 of a monolayer cell density (1.67×10^5 cells/cm^2). Cells were allowed to attach to the glass surface and incubated without perfusion for 75 min

before adding LatA solution. After 45 min from the start of the time-lapse image acquisition, either 5 μM LatA (final concentration) or mock buffer containing only DMSO at concentrations equivalent to that in the LatA solution (<1% v/v) was added. For measurements of population-level oscillation in cells on PLL-coated surface, a 9×9 mm square frame seal (SLF0201, Biorad) was attached to a cover slip (thickness No. 1, 22×22 mm Matsunami) to form a small well. 0.05% PLL solution (P4832, Sigma) was diluted with pure water and poured in or pipetted into the well. The solution was removed after 20 to 30 min. For mock treatment controls, pure water was used instead. After allowing the coverslip to dry at room temperature for 2 to 3 hr, starved cell suspension was 40x diluted to a final volume of 500 μL and plated in the well at approximately 1/4 of a monolayer cell density (1.67×10^5 cells/cm^2). Cells were allowed to attach for 30 min before timelapse imaging.

For single-cell measurements of the cAMP relay response, a syringe containing 1 mL DB was connected to the inlet of the PDMS chip by a tubing. About 50 min before start of the timelapse imaging, the chamber was filled with DB. Starved cell suspension was loaded into the channel to a final density of ~1.3×10^3 cells/cm^2 using a syringe. While allowing the cells to attach to the glass surface for 10 min, a syringe and a tubing filled with DB containing either LatA, LY or an equivalent amount of DMSO as a mock was attached to the Inlet 1 (figure S1*a*) so that the inhibitor was first allowed to equilibriate slowly by passive diffusion for 5 min. The other syringe filled with DB containing cAMP and the inhibitor was attached to Inlet 2 (figure S1*a*). Syringes were placed on a pair of syringe pumps (NE-1002X, New Era Pump Systems). Flow of buffer containing the drug (or mock) from Inlet 1 was initiated 20 min before the start of timelapse acquisition. To realize a step increase in cAMP, the flow rate from Inlet 1 was decreased from 10 to 0 μL/min while that of Inlet 2 was increased from 0 to 10 μL/min. This was achieved by setting the syringe pumps manually at the same time (figure S1*b*).

Measurement of diffusion in agar

To estimate LatA diffusion in the agar plate, the fluorescent intensity profile of a fluorescent marker fluorescein was quantified. The molecular weight of fluorescein is 332 which is close to that of Lat A (MW. 422). A similar time evolution was also

confirmed for Rhodamine B (MW 479) (data not shown). Images of 11.3 cm × 7.5 cm area encompassing the entire 60 mm dish were acquired with an image analyzer (Image Quant LAS 4000, GE Healthcare) using a 460 nm LED as a light source and a filter for green fluorescence (510DF10). Time-lapse images were acquired by 'program mode' with 1 sec exposure time at time interval of 10 min except for the time interval of first and second frame which was 20 min. A 75 μL drop of 10 μM fluorescein in 1% DMSO DB solution was applied to the central reservoir well. The concentration dependence of fluorescent intensity was obtained by preparing 1% agar plates containing fluorescein. Image analysis was performed using ImageJ and Matlab.

Microscopy

Dark-field optics was setup essentially as described earlier [5,6]. Time lapse recordings were performed by taking 1024×1280 pixels 8-bit grey scale images of the plated cells every 30 sec with a CMOS camera (PL-A741, Pixelink) using a program provided by the manufacturer. The center of the acquired images was cropped to a region of 640×640 pixels for analysis.

The microscopy setup for epifluorescent FRET measurements was essentially as described previously[3]. For confocal imaging of cells co-expressing Epac1-camps and Lifeact-mRFPmars, an inverted microscope (IX81, Olympus) equipped with a multipoint scanning unit (CSU-X1, Yokogawa) was employed. 445 nm laser (80 mW, Vortran Laser Technology) and 561 nm laser (75 mW, Melles Griot) were used as light sources in conjunction with a triple-band (445 nm /515 nm /561 nm) dichroic mirror (Yokogawa), conditioning filters for 425 nm (BP425_445HQ, Olympus) and 589 nm (589_BW15, Semrock) and emission filters for 472 nm, 542 nm (Semrock) and 575 nm (BA575, Olympus). A variable neutral density filter was used to reduce the intensity of the excitation light and minimize damage to the cells. The focal plane chosen was at ~2 μm from the coverslip surface, and fluorescence images at CFP, YFP and RFP emission wavelength were acquired sequentially with total delay of less than 300 msec between CFP- and YFP-channels and 2 sec for the RFP-channel. A 20x oil immersion lens (UplanSApo NA 0.85) was used for population-level measurements. A 60x oil immersion lens (PlanApoN NA 1.42, Olympus) were used for single-cell level

observations except for figure S5 where a 100x oil immersion lens (UPlanSApo NA 1.40, Olympus) was employed. Images of 512×512 pixels with 16-bit depth were acquired by a Electron Multiplying CCD camera (CascadeII or Evolve512, Photometrics) at 10 to 30 sec intervals for total durations of 35 min to 150 min using the MetaMorph software (Molecular Devices). At the cell density employed, no more than one cell is typically in a field of view. For confocal imaging, each run of timelapse acquisition was from a single position. For epifluorescent imaging, an automated stage (Sigma Koki) was used to acquire time lapse images from 5 to 6 locations per timelapse recording. The time of image acquisition at the first position is used for the time reference. The delay between the first and the last position was about 7 to 8 sec. All images were acquired at 22 °C. Data were stored as Tagged Image File Format (TIFF) files.

Microfabrication

For single-cell measurements of cAMP relay response, a simple Y-channel chamber was designed using a 2D CAD software (Zunou Rapid, Photron). To obtain a photomask, the CAD file was imported to a direct laser drawing device (DDB-201, Neoark) to irradiate 405 nm laser in a specified pattern on a Cr-deposited photoresist (AZ P1350)-coated mask blank (10×10 cm, CBL4006Du-AZP, Clean Surface Technology). A glass slide (76×26 mm, Matsunami) spin-coated with 50 μm-thick SU-8 3050 (MicroChem) was placed beneath the mask and exposed to ultraviolet light using a mask aligner (MA-20, Mikasa). The irradiated SU-8 was then rinsed in SU-8 developer (MicroChem) to fabricate a mold. The SU-8 mold was used to cast polydimethylsiloxane (PDMS, Sylgard 184 Silicone Elastomer kit, Dow Corning). The thickness of the PDMS chip was ~5 mm. At the edge of each channel, an inlet hole was opened using a 2 mm diameter biopsy punch (BP-20F, Kai Co.). The PDMS chip was then attached to a cover glass (No. 1 thickness, 32×24 mm, Matsunami). After use, the PDMS chip was separated gently from the glass slide, rinsed with ethanol and bonded to a new glass coverslip. The chip was typically reused several times. For PLL-coating, about 10 μL PLL solution (1PLL is 0.5 mg/mL) was introduced from the inlet and the solution was removed after 0.5 hr. The chamber was dried for > 2 hrs at room temperature and used on the same day. The coated chip was used only once.

Supplementary Figures

Supplementary Figure S1

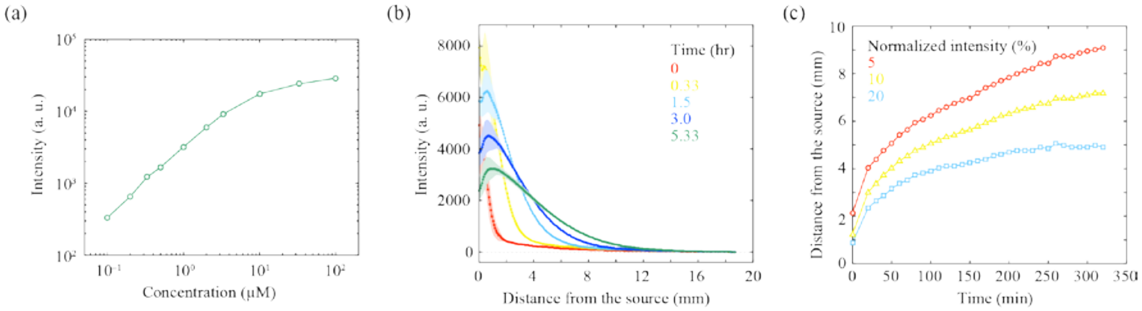

Figure S1. An estimate of diffusion in the agar plate. (*a*) A dose-curve of the marker fluorescein used as a surrogate for LatA. The fluorescence intensity of an agar plate without fluorescein was subtracted as a background from the raw data. (*b*) The radial profile of fluorescent intensity from the edge of the reservoir well. The mean intensity and the standard deviation at $t = 0, 0.33, 1.5, 3.0,$ and 5.33 hr. (*c*) The temporal change in the position at the cut-off threshold 5% (red circle), 10% (yellow triangle), and 20% (blue square) of the source fluorescent intensity (10 µM fluorescein at time 0 hr). Regions at 3 to 5 mm from the source is exposed to approximately 20% of the source concentration between 1 to 4 hrs. By 5.5 hrs, an area ~ 7 mm from the source (equivalent to the field of view in Fig. 1*a*) will be covered with 10% of the source concentration (yellow plots).

Supplementary Figure S2

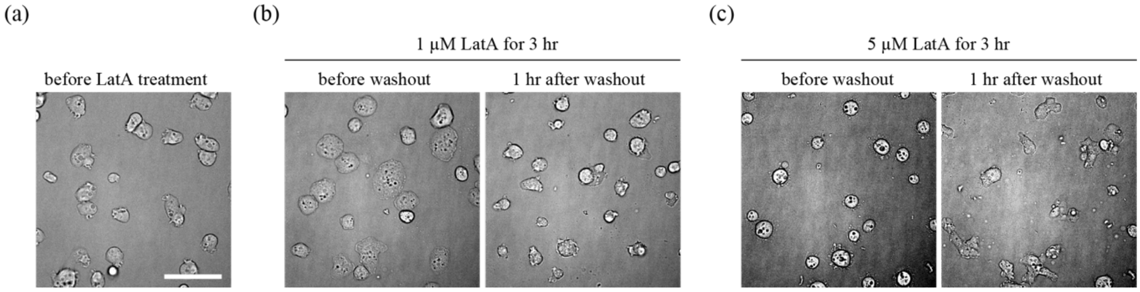

Figure S2. Cells under prolonged LatA treatment recovered random motility after washout. 2.5hrs starved cells plated on a glass bottom dish (MatTek) (*a*) were treated with 1 μM (*b*) or 5 μM LatA (*c*) for 3 hr. Snapshots immediately before the washout (left panels) and 1hr after LatA washout (right panels) are shown for comparison of cell shape. LatA was removed manually by repeating 1:10 dilution of the supernatant with fresh DB for 5 times. The scale bar is 50 μm.

Supplementary Figure S3

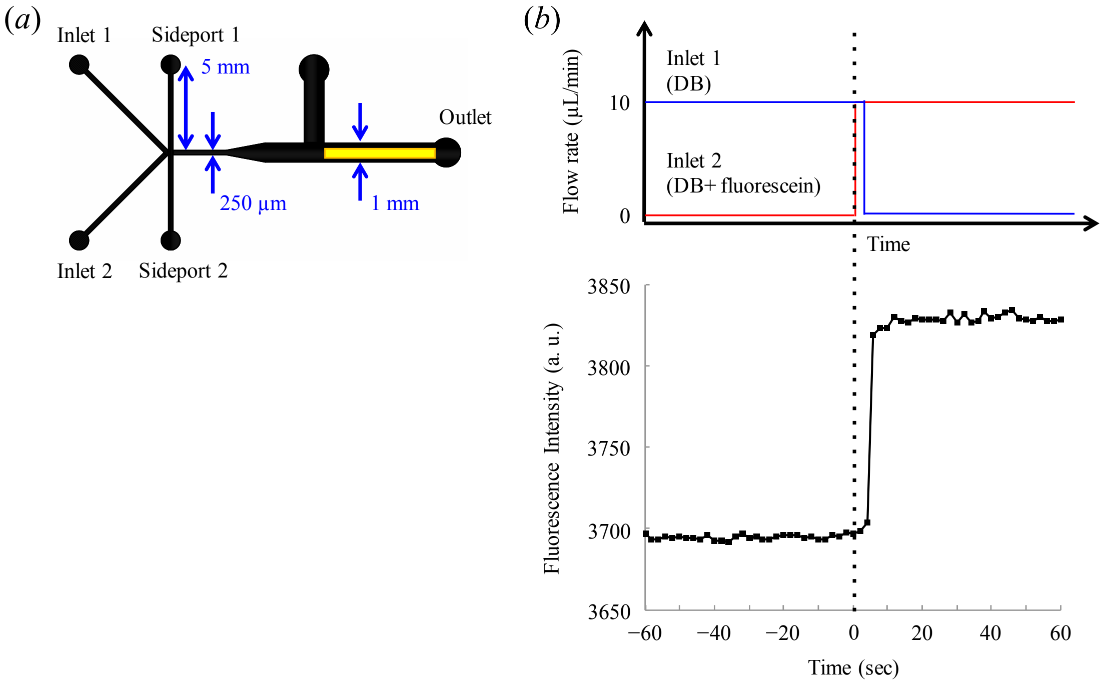

Figure S3. Microfluidics setup used to deliver a step increase in extracelluar cAMP. The small working volume allows rapid exchange of buffer and minimizes the amount of inhibitor compounds required in perfused flow. (a) The chamber design. The chamber (inner height 50 µm) was made of PDMS and attached to a coverslip (see Supplementary Methods). The cells were loaded from the Inlet 1. An area indicated in yellow is the observation area for timelapse imaging. (b) A time course of stimulus application in the microfluidics chamber. DB and DB containing 1 µM fluorescein in DB was perfused at the rate of 10 µL/min from the Inlet 1 and 2 respectively. The flow rate was switched at time 0. The mean fluorescent intensity obtained from the center of the chamber (1×5 mm) region using confocal microscopy. The buffer exchange completes in approximately 2 seconds. The exact same scheme was employed to deliver cAMP.

Supplementary Figure S4

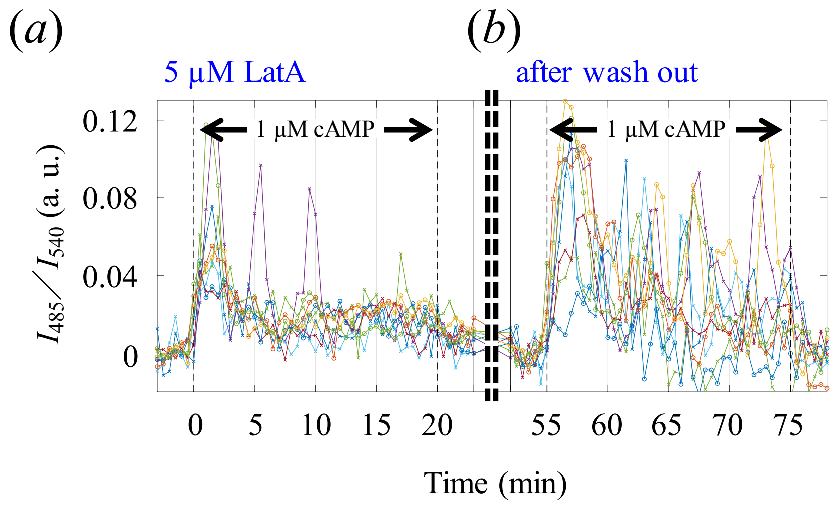

Figure S4. Single-cell level cAMP relay response in cells treated with 5 μM LatA was restored after drug removal. (*a*) cAMP relay response in cells treated with 5 μM LatA. (*b*) cAMP relay response after washing with DB with DMSO for ~20 min. Same cells were tracked before and after washing. The colors specify each individual cells.

Supplementary Figure S5

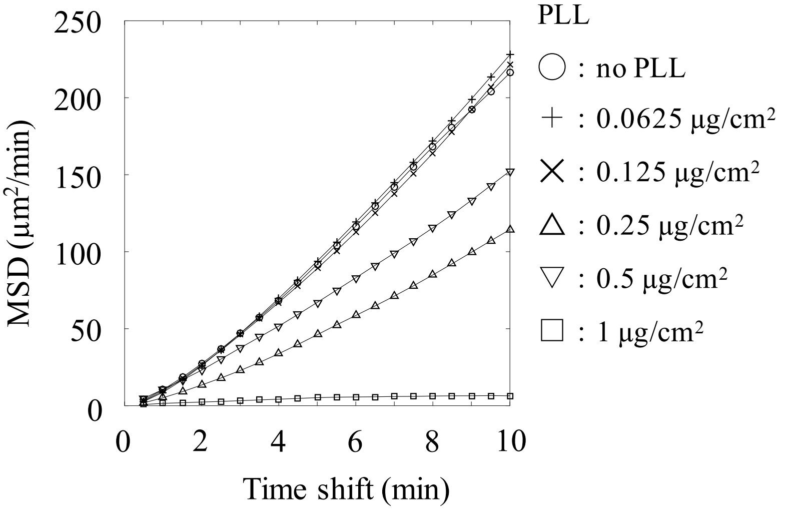

Figure S5. Random cell motion was impaired in cells attached to PLL-coated coverslips. 4 hrs starved cells were suspended in DB and plated at approximately 5×10^{3} cells/cm^{2} on a coverslip coated with indicated amount of PLL. A frame seal was used to form a chamber (see Supplementary Materials and Methods). Fluorescent images of Epac1-camps were taken at 30 sec intervals for 60 min using a 20x objective lens. The cell centroids were manually tracked and their mean square displacement was calculated (n = 13, 15, 9, 29, 22, 3 for PLL density of 0, 0.062, 0.125, 0.25, 0.5 and 1.0 µg/cm^{2}).

Supplementary Figure S6

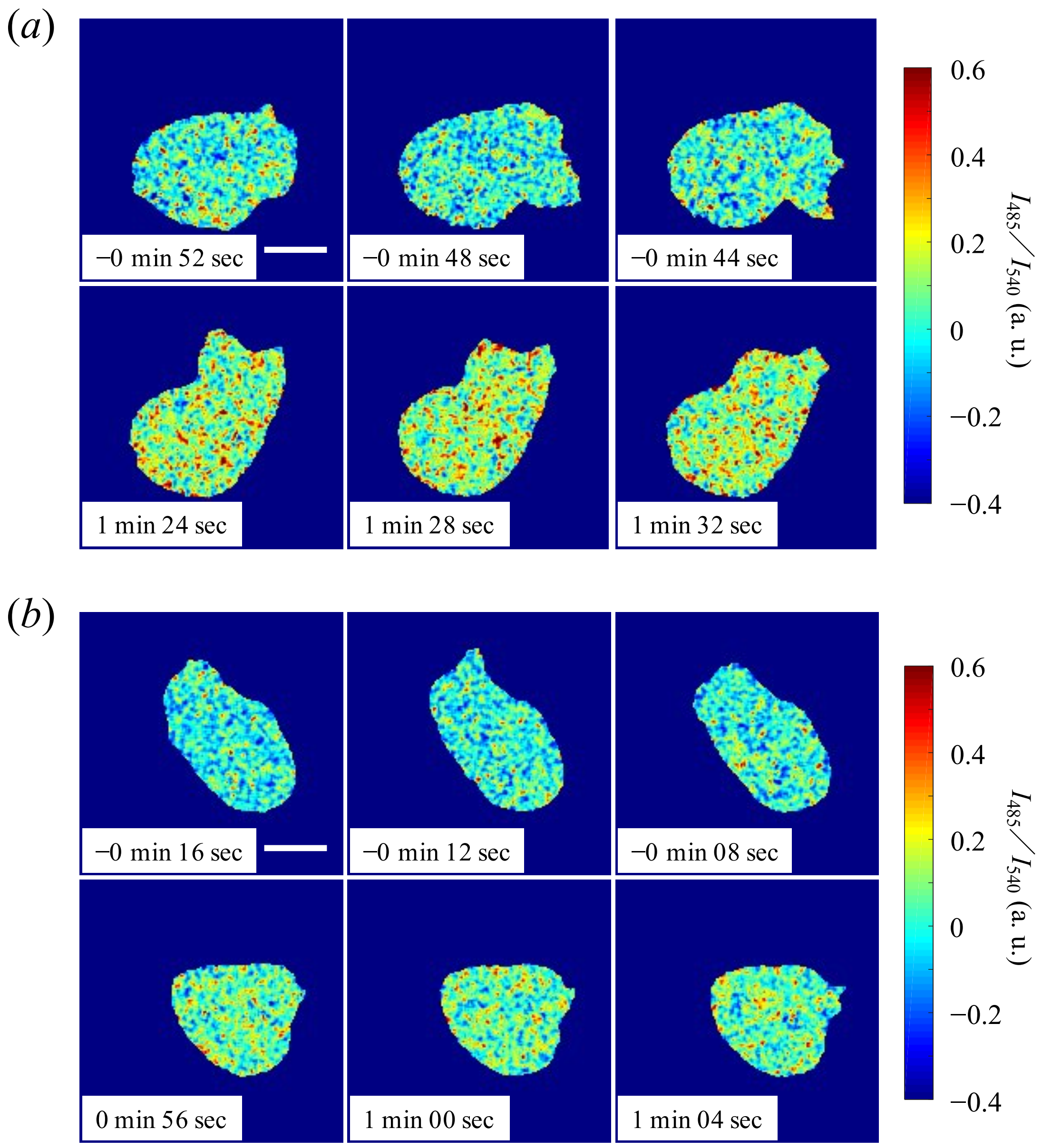

Figure S6. Confocal sections of Epac1camps/AX4 cells stimulated with 10 nM cAMP. (*a*, *b*) Snapshots from two representative timelapse data. Perfusion flow was switched from DB to DB containing cAMP at $t = 0$ sec. Images were taken at 4 sec intervals using 100x objective lens. Scale bars are 5 μm.

Supplementary Figure S7

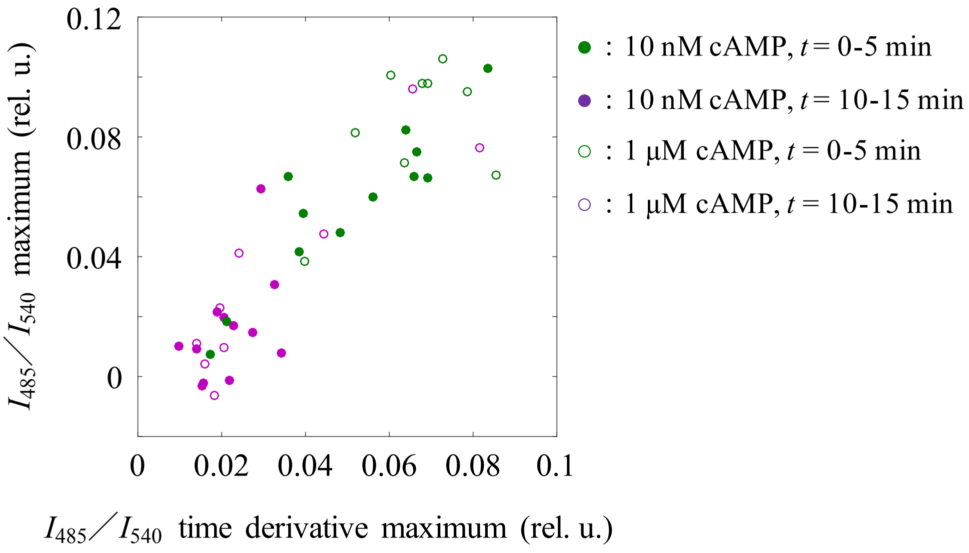

Figure S7. Scatter plots between the maximum of the FRET signals and the rate of their increase. Data were taken from the confocal image used to plot figure 6*b* and *c*. The difference between consecutive frames (time step 10 sec) was calculated and its maximum in the respective time windows were plotted. Pearson correlation coefficient $r = 0.8984$.

Supplementary References

1. Taniguchi, D., Ishihara, S., Oonuki, T., Honda-Kitahara, M., Kaneko, K. & Sawai, S. 2013 Phase geometries of two-dimensional excitable waves govern self-organized morphodynamics of amoeboid cells. *Proc Natl Acad Sci USA* **110**, 5016–5021. (doi:10.1073/pnas.1218025110)

2. Lemieux, M. G., Janzen, D., Hwang, R., Roldan, J., Jarchum, I. & Knecht, D. A. 2014 Visualization of the actin cytoskeleton: different F-actin-binding probes tell different stories. *Cytoskeleton (Hoboken)* **71**, 157–169. (doi:10.1002/cm.21160)

3. Gregor, T., Fujimoto, K., Masaki, N. & Sawai, S. 2010 The onset of collective behavior in social amoebae. *Science* **328**, 1021–1025. (doi:10.1126/science.1183415)

4. Nellen, W., Silan, C. & Firtel, R. A. 1984 DNA-mediated transformation in Dictyostelium discoideum: regulated expression of an actin gene fusion. *Mol Cell Biol* **4**, 2890–2898.

5. Sawai, S., Thomason, P. A. & Cox, E. C. 2005 An autoregulatory circuit for long-range self-organization in Dictyostelium cell populations. *Nature* **433**, 323–326. (doi:10.1038/nature03228)

6. Sawai, Guan, Kuspa & Cox 2007 High-throughput analysis of spatio-temporal dynamics in Dictyostelium. *Genome Biol* **8**, R144. (doi:10.1186/gb-2007-8-7-r144)
